# Autotrophic vs. Heterotrophic Cultivation of the Marine Diatom *Cyclotella cryptica* for EPA Production

**DOI:** 10.3390/md19070355

**Published:** 2021-06-23

**Authors:** Adelaide Cupo, Simone Landi, Salvatore Morra, Genoveffa Nuzzo, Carmela Gallo, Emiliano Manzo, Angelo Fontana, Giuliana d’Ippolito

**Affiliations:** 1 Institute of Biomolecular Chemistry ICB-CNR, Via Campi Flegrei 34, 80078 Naples, Italy; adelaide.cupo@gmail.com (A.C.); s.morra@icb.cnr.it (S.M.); nuzzo.genoveffa@icb.cnr.it (G.N.); carmen.gallo@icb.cnr.it (C.G.); emanzo@icb.cnr.it (E.M.); afontana@icb.cnr.it (A.F.); 2 Department of Biology, University of Naples “Federico II”, Via Cinthia, 80126 Napoli, Italy; simone.landi@unina.it

**Keywords:** microalgae, omega-3 fatty acids, biomass, lipids

## Abstract

Recently, the marketable value of ω-3 fatty acid, particularly eicosapentaenoic acid (EPA), increased considering their health effects for human consumption. Microalgae are considered a valuable and “green” source of EPA alternative to fish oils, but considerable efforts are necessary for their exploitation at an industrial level. Due to the high operation costs of photoautotrophic microalgae cultivation, heterotrophic growth represents a promising economic solution. Marine diatoms are the major ecological producers of ω-3 fatty acids. Few species of diatoms are capable to grow in the dark using organic carbon sources. The marine diatom *Cyclotella cryptica* was cultivated for 14 days under photoautotrophic and heterotrophic conditions to define the effects on growth parameters, lipid production, total fatty acids and EPA content. Photoautotrophic conditions led to a total EPA production of 1.6% of dry weight, 12.2 mg L^−1^ culture and productivity of 0.9 mg L^−1^ day^−1^. The heterotrophy cultures reported a total EPA production of 2.7% of dry cell weight, 18 mg L^−1^ culture, a productivity of 1.3 mg L^−1^ day^−1^, which are promising values in the prospective of improving culture parameters for the biotechnological exploitation of dark cultivation. *C. cryptica* could be a potential candidate for the heterotrophic production of EPA, also considering its robustness, capacity to resist to bacterial contaminations and plasticity of lipid metabolism.

## 1. Introduction

Eicosapentaenoic acid (EPA) represents a central nutrient for human consumption to counteract cardiovascular disease, diabetes, different types of carcinoma, diabetes mellitus and brain disorders [1,2,3]. EPA is also a key nutritional requirement during childhood, improving cognitive and visual development. EPA, as well as docosahexaenoic acid (DHA), cannot be synthesized by human beings due to lack of some desaturases and elongases that take part in the synthesis of EPA and DHA from parent ω-3 fatty acids such as linolenic acid (C18:3 ω-3) [4]. To fulfill the daily intake requirement, these ω -3 fatty acids must be taken from outside diet sources [5]. Actually, ω-3 polyunsaturated fatty acids (PUFA) were mainly obtained from fatty fish species, such as herring, mackerel, sardine, menhaden and salmon [6]. However, the global fish stocks cannot be considered a sustainable source of ω-3 fatty acids to fulfill the ever-rising global demand. To eliminate the issues related with fish oils, the exploration of alternative resources of PUFA has been gaining interest in recent years. A total annual worldwide demand of PUFA was calculated at over 1.27 million tonnes [7]. In 2019, the global ω-3 market size was evaluated at USD 2.49 billion and a 7% increase is expected over the period 2020–2027 [8].

Many marine microalgae species naturally produce EPA and DHA as components of glycerolipids [9,10], thus resulting in a sustainable source of these fatty acids [11,12].

Microalgae were mainly cultivated in phototrophic conditions in different systems, namely, open ponds, flat panel or photobioreactors [13]. Although many attempts to develop biorefinery platforms to obtain multiple products from microalgae (oils, pigments, proteins and carbohydrates), the operation costs remain non-competitive in comparison to other technologies [14]. At present, algal oil represents less than 2% of human EPA/DHA consumption, but its contribution has been increasing due to several social attributes including its environmental friendliness, the absence of ocean borne contaminants, its vegetarian nature, as well as the possibility to be manufactured under kosher or halal conditions. A feasible alternative for phototrophic cultures is the use of their heterotrophic growth capacity in the absence of light. Heterotrophy requires an organic carbon source dissolved in the culture media to replace the fixation of atmospheric CO_2_ [15]. Any fermenter or bioreactor can be employed for this purpose, such as those used for the industrial production of medicines, beverages, food additives and energy. This approach represents a major outcome in the reduction in microalgae cultivation costs.

A limited number of microalgae species can grow in heterotrophic conditions, especially *Chlorella protothecoides*, *Galdieria sulphuraria*, *Nitzchia laevis*, *Crypthecodinium cohnii* and *Neochloris oleoabundans* [16]. These species were mainly used for the biotechnological production of astaxanthin, biomass, DHA, EPA, hydrogen, lipids, lutein and phycocyanin [14]. The identification of candidate species and strains that have a high potential to grow in heterotroph, as well as the improvement of culture conditions, represent a major challenge for biotechnology research [17,18].

Among microalgae, diatoms represent a primary trophic source in the marine food chain, sustaining zooplankton and fish nutrition and therefore are the major ecological producers of ω-3 fatty acids [19]. Lipid accumulation in these microorganisms represent a carbon and energy storage mechanism, mainly occurring upon perturbing conditions or when photosynthesis exceeds the limitations of growth [9,20,21]. The lipid content can range from 10 to 60% of the diatom biomass and this value is influenced by the species and the metabolic status of the cells [22,23]. Despite the recognized role of diatoms as cell factories for high-value products, few studies have been explored their capacity to grow under heterotrophic conditions. *Nitzschia laevis* represents the most studied case for which impact of cultivation parameters on heterotrophic production of EPA has been assessed [24,25], suggesting that this diatom is a good heterotrophic EPA producer.

The marine diatom *Cyclotella cryptica* has been reported as a species capable of heterotrophic growth [26,27,28], that has been trialed as part of an artificial diet in the development of juvenile mollusks [29]. Subsequent works have analyzed biochemical composition and nutritional aspects of *C. cryptica* at the exponential growth phase (maximum of 4 days of cultivation), as continuous cultivations are frequently used in aquaculture facilities [30,31,32], or as fucoxanthin producers [33]. The aim of this study is the assessment of growth performances and EPA production in *C. cryptica* under photoautotrophic and heterotrophic conditions.

## 2. Results and Discussion

### 2.1. Growth Curves and Biomass Production by Cyclotella cryptica

**Figure 1:**
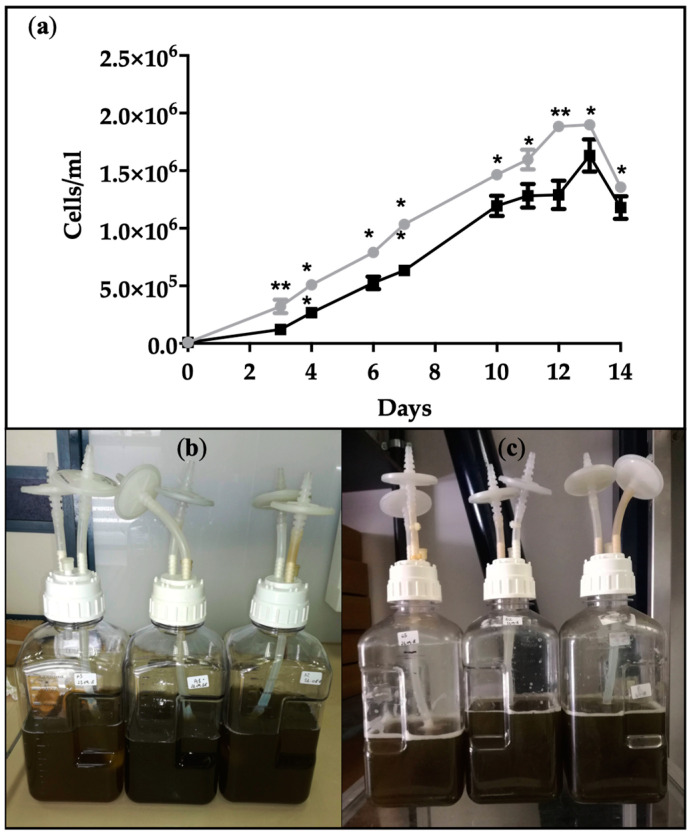
(**a**) Growth curves of *C. cryptica* cells cultivated in autotrophic (grey line) and heterotrophic (black line) conditions. Asterisks indicate significantly different values in autotrophic and heterotrophic samples at *p* ≤ 0.05 (*) and *p* ≤ 0.001 (**). Data are expressed as means ± SD, *n* = 3. *C. cryptica* cultures in 2 L propylene carboys under autotrophic (**b**) and heterotrophic (**c**) conditions.

The marine diatom *Cyclotella cryptica* was grown in autotrophic and heterotrophic conditions in order to compare the growth performances and EPA production among ω-3 polyunsaturated fatty acids. Autotrophic cultivation was guaranteed by an illumination at 200 µmol (photons) m*^−^*^2^ s*^−^*^1^ with a 14:10 h (light/dark) photoperiod, whereas heterotrophic growth was carried out by cultivating cells completely in the dark and by supplementing the medium with glucose as an organic carbon source. As showed in Figure 1a, growth curves under autotrophic and heterotrophic conditions displayed a similar trend under a sufficient nutrient regime, albeit the cellular density was slightly smaller in the dark. After a short adaptation phase to the heterotrophic conditions of 3*–*4 days, the growth rate reflected a rapid increase in cell division. In fact, the doubling time was 2 ± 0.05 days in heterotrophy in comparison with a value of 3.3 ± 0.2 days in autotrophy, supporting the concept that a minor amount of time is necessary to double the cell number under heterotrophy. The cultures maintained the same intense brown color under photoautotrophic and heterotrophic conditions (Figure 1b,c), due to the persistent presence of fucoxanthin [33].

### 2.2. Biomass and Lipid Production

**Figure 2:**
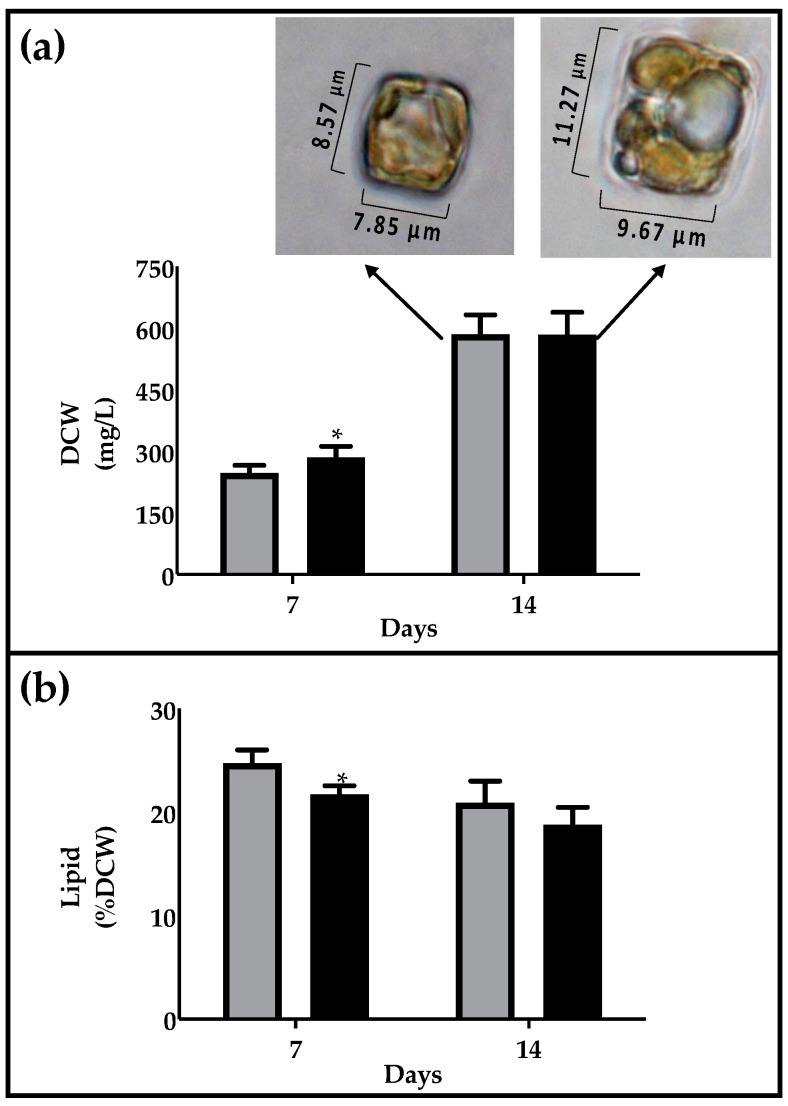
Biomass and lipid content in *C. cryptica* grown in autotrophic (grey bars) and heterotrophic (black bars) conditions. (**a**) Dry cell weight (DCW) expressed as mg/L; boxes contained microscopy images of cells. (**b**) Lipid content expressed as % of DCW. Data are expressed as means ± SD, n = 3. Asterisks indicate significantly different values in autotrophic and heterotrophic samples at *p* ≤ 0.05 (*).

Biomass production was evaluated on the 7th day (exponential phase) and 14th day (first point of senescent phase). After 7 days, the dry biomass was 246.4 ± 15.9 mg L*^−^*^1^ in autotrophy and 285.5 ± 21.3 mg L*^−^*^1^ in heterotrophy, and similar values of around 584 ± 39 mg L*^−^*^1^ were reached after 14 days in both conditions (Figure 2a). Although major cell densities were reached under autotrophic conditions, the comparable biomass level can be explained by considering the major dimension of heterotrophic cells, showing a medium area of about 110 ± 4 µm^2^, in comparison with 64 ± 9 µm^2^ in autotrophy (Figure 2a), according to the evidence that the glucose generally induced an increase in cell weight due to the higher energetic content of this carbon source. The data are in agreement with reports in other microalgae species such as *Galdiera sulphuraria*, in which a major biomass production and average cell size (10*–*30%) has been described in heterotrophic conditions [34]. Response in biomass production is strongly species dependent as demonstrated for *Chlorella protothecoides*, *C. vulgaris* and *C. sorokiniana* [35,36]. The marine diatom *Nitzschia laevis* produces major biomass under heterotrophic conditions [25], although the photoautotrophic performance depends on the illumination and photoperiod used for comparation, as well as whether inoculum was cultured in the dark or in the light [21,37].

The lipid percentage was not significantly affected under two different culture conditions, being 22.2 and 18.8% of dry cell weight (DCW) in autotrophy and heterotrophy, respectively (Figure 2b). Cells were cultivated in both cases under a sufficient nutrient regime and were provided with adequate glucose input in the case of dark cultivation. The response in lipid accumulation by the microalgal cells under autotrophic, mixotrophic and heterotrophic conditions is dependent on the strain and on operational cultivation parameters, such as nutritional factors [38]. *Nannochloropsis gaditana* showed similar percentages of lipids on dry weight in autotrophic, mixotrophic and heterotrophic conditions (10.7*–*15.7%) [37]. *Chlorella* species showed a higher lipid content in heterotrophy, in comparison to autotrophic conditions [36].

### 2.3. Fatty Acid Composition

Fatty acid methyl ester (FAME) analysis using GC–MS showed a simplified framework in heterotrophy (Table 1), with a general reduction in some polyunsaturated fatty acid species. Interestingly, the main plastidial polyunsaturated diatom markers 16:2 ω-4, 16:3 ω-4 and 16:4 ω-1 were significantly reduced or disappeared under heterotrophic conditions, in favor of monounsaturated 16:1 ω-7, which is the most abundant fatty acid (around 60% of total fatty acids). EPA and DHA, which remain the most abundant fatty acids among the ω-3 pool, slightly decreased in dark growth, representing 15% and 1.6% of total fatty acids (TFA), respectively, at day 14. Interestingly, 18:0 completely disappeared under heterotrophic conditions.

Similar results were obtained after 7 days of cultivation, supporting the concept that the establishment of dark lipid-phenotype occurs early and is maintained along the growth curve. Data about the EPA% on TFA at the 14th day are in agreement with previous works on the diatoms *C. cryptica* and *N. laevis* grown in heterotrophy, reporting a range of 15*–*20%, depending on the salinity and silicate levels, glucose input and temperature [31,39]. Overall, the percentage of saturated fatty acids (SFA) did not significantly change, whereas monounsaturated fatty acids (MUFA) significantly increased at the expense of polyunsaturated fatty acids (PUFA), arising values around 60%. This massive mutual redistribution among MUFA and PUFA comparing autotrophy vs. heterotrophy has been rarely documented in marine diatoms, whereas some studies reported a similar, but less attenuated, effect in microalgae such as *Chlorella zofingiensis* and *Galdiera* sp. [40,41]. *Chlorella vulgaris* showed an opposite trend, with a decrease in MUFA in favor of PUFA in heterotrophy, suggesting that the response of fatty acid elongation and desaturation strictly depends on the microalgal species [42,43,44].

### 2.4. EPA Production and Productivity

The ERETIC ^1^H-NMR method was used for the quantitative assessment of Total Fatty Acids (TFA) and ω-3 fatty acids in organic extracts, by integrating the diagnostic peaks at 2.35 ppm (methylene protons in *α* to carbonyl group) and at 0.9 ppm (methyl protons of ω-3 fatty acids), respectively [45].

**Figure 3:**
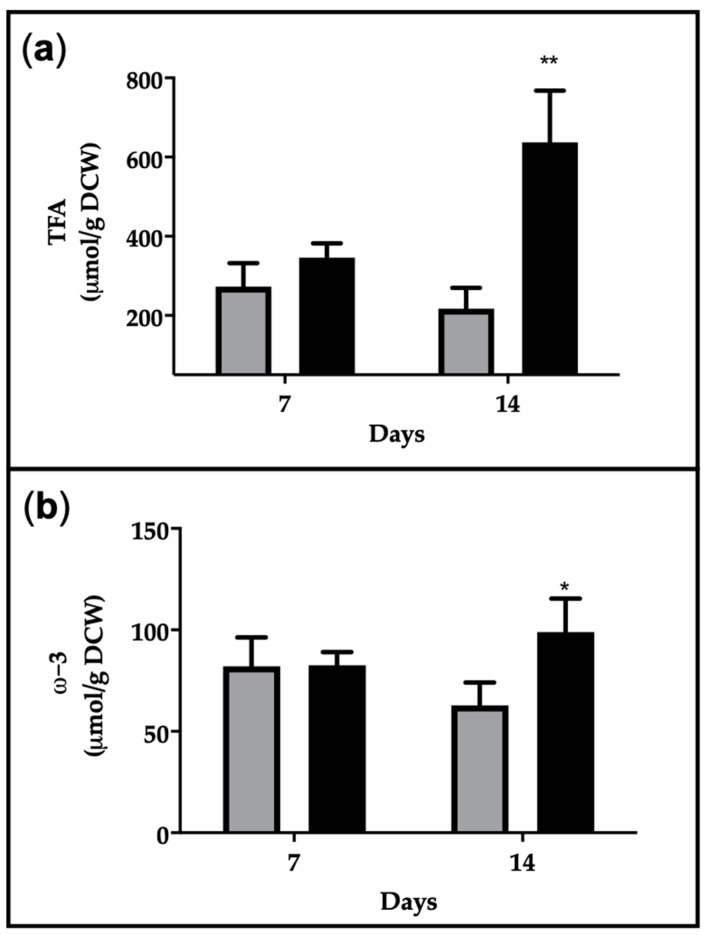
(**a**) TFA production and (**b**) ω-3 production assessed by ^1^H-NMR in *C. cryptica* cultivated in autotrophic (grey bars) and heterotrophic (black bars) conditions. Data are expressed in µmol/g of Dry Cell Weight (DCW) as means ± SD. Asterisks indicate significantly different values in autotrophic and heterotrophic samples at *p* ≤ 0.05 (*) and *p* ≤ 0.001 (**).

In heterotrophy TFA were not significantly different after 7 days of cultivation but were 3-fold higher in heterotrophy than autotrophy after 14 days, arising values of 630 µmol/g DCW (Figure 3a). This is in agreement with the morphology of the cells, which showed an increased presence of vacuolar structures in the dark, attributable to oil droplets (Figure 2a). The diatom *N. laevis* also showed an increase in TFA in the heterotrophic growth mode in comparison to photoautotrophic conditions [25]. The ω-3 fatty acid content is around 80 µmol/g DCW in all conditions, except in the dark at day 14, in which it arises values of 100 µmol/g DCW (Figure 3b).

Considering EPA contribution to ω-3 fatty acids determined by GC–MS, the quantitative assessment of EPA production is reported in Table 2. The results indicated no significant differences in the EPA production at day 7 between autotrophy and heterotrophy, but major values were reached at day 14 in heterotrophy, being 2.7% of DCW, 18 mg L*^−^*^1^ and productivity of 1.3 mg L*^−^*^1^ day*^−^*^1^.

Despite the recognized role of diatoms as cell factories for high-value products, few studies have explored their capacity for growth under heterotrophic conditions. *Nitzschia laevis* represents the most studied case for which the impact of cultivation parameters on the heterotrophic production of EPA has been assessed [25,38] suggesting that this diatom is a good heterotrophic EPA producer.

Among the numerous marine diatoms screened, few species were able to grow in heterotrophy and were investigated for heterotrophic EPA production, e.g.*, N. laevis*, *Navicula incerta*, *Navicula pelliculosa* and *C. cryptica* (Table 3). Although culture volumes and duration are often limited, the EPA content was estimated between 0.5 and 2.7% of DCW, and EPA represents an abundant polyunsaturated fatty acid, with values from 4.6 to 23.2% of TFA.

Glucose and silicate input play a critical role in the production of EPA and TFA in marine diatoms. Although a sufficient amount of silicate appears necessary for an adequate carbon metabolism, the glucose concentration appeared to be mainly related to TFA amounts and, in minor part, to the EPA percentage. Major glucose concentrations (20*–*50 g/L) led to a major accumulation of TFA but did not change the EPA% on TFA. The nutritional factors to significantly burst EPA biosynthesis have not yet been determined under heterotrophic conditions. Some environmental factors (e.g., salinity, temperature, pH, nitrate sources, silicate concentration), which can modulate lipid metabolism in a massive way under autotrophic conditions, did not affect the fatty acid distribution under heterotrophic growth. Alternative cultivation strategies to improve the final yield of biomass and EPA have been developed. A combination of perfusion and bleeding systems enhance the ability of *N. laevis*, obtaining 6.75 g L*^−^*^1^ day*^−^*^1^ and 175 mg L*^−^*^1^ day*^−^*^1^ of biomass and EPA yield, respectively, which are among the highest values ever reported in microalgal cultures [46].

Due to the high biotechnological potential of marine diatoms, the comprehension of lipid metabolism and fatty acid biosynthesis under heterotrophic conditions is crucial to develop economically sustainable processes for the production of high-added value products.

## 3. Conclusions

Nowadays, the efficient heterotrophic cultivation of diatoms still remains an open challenge. The present work contributed to the expansion of the knowledge about the “dark phenotype” of *C. cryptica* obtained under heterotrophic conditions, in comparison with the classic profile of diatoms under autotrophic conditions. The results indicated comparable levels of EPA production, suggesting that light and CO_2_ can be substituted by dark and an organic C source, with the view of reducing cultivation costs using fermentation technologies. This is a promising outcome to ulteriorly improve the heterotrophic cultivation of *C. cryptica* as a suitable method for EPA production to face the increasing global EPA demand. Further studies will be necessary to elucidate the biochemical and molecular network regulating the adaptation of marine diatoms to dark, the metabolic pathways for EPA biosynthesis and lipid remodeling.

## 4. Materials and Methods

### 4.1. General

All solvents and standards were purchased from Sigma-Aldrich (Milan, Italy).

^1^H NMR spectra were recorded on a Bruker DRX 600 spectrometer (purchased from Bruker, Milan, Italy) equipped with an inverse TCI CryoProbe. Peak integration, ERETIC measurements and spectrum calibration were obtained by the specific subroutines of the Bruker Top-Spin 3.1 program. Spectra were acquired with 14 ppm of spectral width (8417.5 Hz), 32 K of time domain data points, 90° pulse, 32 K spectrum size and processed with 0.6 Hz of line broadening for the exponential decay function.

### 4.2. Strain and Culture Conditions

*Cyclotella cryptica* (CCMP 331) was purchased from National Center for Marine Algae and Microbiota (Bigelow Laboratory for Ocean Sciences, East Boothbay, ME, USA) and was maintained in an F/2 medium. *C. cryptica* was grown at 20 ± 2 °C in 2 L of polycarbonate carboy in 2 L of pre-filtered sterile (0.22 μm) f/2 medium [47] at an initial concentration of 1 × 10^4^ cells mL^−1^. Cultures were gently bubbled with sterile air. In autotrophic conditions, diatoms were grown under artificial light (200 μmol photons m^−2^ s^−1^), provided by daylight fluorescent tubes (OSRAM 965, Germany) with a 14:10 h light:dark photoperiod. In heterotrophic conditions, cultures were incubated in the dark and f/2 medium was supplemented with two pulses of 1g/L glucose for each one, at day 0 and day 7. Macronutrient levels were maintained at high regimes, adding nitrates (NaNO_3_, 882 µmol L^−1^) and phosphates (NaH_2_PO_4_·H_2_O, 36 µmol L^−1^) every 48 h, and silicate (Na_2_SiO_3_·9H_2_O, 107 µmol L^−1^) every 24 h. Cell growth was monitored using a microscope (Axio VertA1, Carl Zeiss, magnification of 20X, Milan, Italy) and a Bürker counting chamber (depth 0.100 mm, Merck, Leuven, Belgium).

### 4.3. Calculation

Growth rate of cultures µ, expressed as divisions day^−1^, was calculated according to Equation (1), as follows:

µ = (ln(N_2_) − ln(N_1_))/t_2_ − t_1_ (1)

where N_1_ and N_2_ are cell numbers (cells/mL) at time 1 (t_1_) and time 2 (t_2_) at the extremes of the linear phase [48]. Doubling time t_d_ was calculated according to Equation (2), as follows:

t_d_ = ln2/µ (2)

Number of doublings (n) at a time interval t is determined by the relation t/t_d_, where td is the doubling time or time required to achieve a doubling of the number of viable cells.

### 4.4. Biomass Content

Cells (500 mL) were harvested using centrifugation with a swing-out rotor at 2300 g for 10 min (Allegra X-12R, Beckman Coulter Inc., Palo Alto, CA, USA). Pellets were frozen at −80°C and lyophilized with a MicroModulyo 230 (Thermo Electron Corporation, Milford, MA, USA). Dry weight was estimated on lyophilized biomass and expressed as mg L^−1^ culture. Biomass productivity was calculated in agreement with d’Ippolito et al. [9].

### 4.5. Lipid Extraction

Lipid extraction was performed using the Methyl tert-butyl ether (MTBE) method, in agreement with Cutignano et al. [10], using 4,4′-Dihydroxybenzophenone (DHBP) (1 mg mL^−1^) as the internal standard. In particular, dry cell pellet (50 mg) was suspended with 500 µL of DHBP and 400 µL of MeOH. After vortexing, 3 mL of MTBE were added to allow extraction at room temperature for 1 h, at constant shaking. Then, 750 µL of water were added and samples were left for another 10 min at room temperature, under shaking. Organic extracts were recovered (upper phase), after centrifugation at 1000× *g* for 10 min. The lower phase was re-extracted with 1 mL of MTBE and 750 µL of water, after centrifugation the upper phase was combined with the previous one. The extract was dried under nitrogen flow and weighed to gravimetrically estimate the lipid content (mg L^−1^ culture).

### 4.6. NMR Analysis of Lipid Extracts

Crude microalgal extracts were dissolved in 700 µL of CDCl_3_/CD_3_OD 1:1 (*v*/*v*) and transferred to a 5-millimeter NMR tube. ^1^H-NMR spectra were recorded on a Bruker DRX 600 spectrometer equipped with an inverse TCI CryoProbe. Chemical shift was referred to CHD_2_OD signal at δ 3.34. Quantitative assessment of fatty acids was established using the ERETIC method in agreement with Nuzzo et al. [45]. The ERETIC signal was calibrated on the doublet signal at δ 6.90 of DHBP (2.23 µmol in 700 µL of CDCl3/CD3OD 1:1). Peak integration, ERETIC measurements and spectrum calibration were obtained by the specific subroutines of Bruker Top-Spin 3.1 program. Spectra were acquired with 14 ppm of spectral width (8417.5 Hz), 32 K of time domain data points, 90° pulse, 32 K spectrum size and processed with 0.6 Hz of line broadening for the exponential decay function.

The diagnostic peaks in the region between 2.38 and 2.28 ppm, centered at 2.35 ppm (methylene protons in *α* to carbonyl group), and between 0.99 and 0.95, centered at 0.97 ppm (methyl protons of ω-3 fatty acids) were integrated to assess the µmol of Total Fatty Acids (TFA) and ω-3 fatty acids, respectively [45].

### 4.7. GC–MS Analysis of Lipid Extracts

The total fatty acid composition of organic extracts was determined using GC–MS on the corresponding fatty acid methyl esters (FAMEs) obtained after saponification of lipid extracts with Na_2_CO_3_ in methanol at 40 °C for 4 h. The reaction mixture was diluted with milliQ water (to dissolve completely Na_2_CO_3_), neutralized with HCl 1M, and extracted with diethylether three times. Combined organic extracts were dried under a nitrogen stream, dissolved in MeOH at a final concentration of 1 µg µL^−1^ and analyzed using GC–MS (Thermo Focus GC Polaris Q) equipped with an ion-trap, EI (70 eV), a 5% diphenyl column, an injector temperature of 210 °C, a transfer line temperature of 280 °C. Elution of free fatty acid methyl esters required an increasing gradient of temperature according to the following method: 160 °C for 3 min, increase by 3 °C/min up to 260 °C, increase by 30 °C/min up to 310 °C, 7 min at 310 °C. FAMEs have been identified by the comparation of retention time and mass spectra with FAMEs’ standard mixture (Marine source analytical standards, Sigma Aldrich, Milan, Italy). Fatty acid content was expressed as a percentage of total fatty acids, according to Equation (3), as follows:

% FA= (Area _FA_ * 100)/Area _sum of all FA_ (3)

### 4.8. Assessment of EPA Productivity

EPA content on total ω-3 (X_EPA_) was established using the GC–MS integrating area of all ω-3 fatty acids (EPA; DHA; 18:3 ω-3 and 18:4 ω-3), according to Equation (4). EPA (%DCW) was assessed according to Equation (5), considering the contribution of EPA fraction to ω-3 mmol assessed using ^1^H-NMR on organic extract obtained from 50 mg of DCW of EPA. EPA production was calculated by considering EPA expressed as % DCW, according to Equation (6), as follows:

X _EPA/ω-3_ = Area _EPA, GC–MS/_Area _sum all ω-3, GC–MS_ (4)

EPA _(%DCW)_ = (X_EPA/ω-3_ × mmol ω-3 _1H-NMR_ × PM _EPA_)/50 mg DCW (5)

EPA production _(mg/L culture)_ = (Biomass _(mg/L)_ × 100)/EPA _(%DCW)_ (6)

### 4.9. Statistics

Each experiment was made in at least three replicates. Values were expressed as mean ± standard deviation (SD). The statistical significance was evaluated through Student’s *t*-test (*p* ≤ 0.05).

## Figures and Tables

**Table 1:** Fatty acid composition of *C. cryptica* grown under autotrophic and heterotrophic condition for 7 and 14 days, expressed as a percentage of Total Fatty Acids (TFA). SFA = Saturated Fatty Acids; MUFA = MonoUnsaturated Fatty Acids; PUFA = PolyUnsaturated Fatty Acids, expressed as a percentage of TFA. Asterisks indicate significantly different values in autotrophic and heterotrophic samples at *p* ≤ 0.05 (*) and *p* ≤ 0.001 (**). Data are expressed as means ± SD, n = 3.

	Day 7		Day 14	
Fatty Acids	Autotrophy	Heterotrophy	Autotrophy	Heterotrophy
14:0	5.3 ± 0.1	2.9 ± 0.5 *	5.5 ± 0.03	2.5 ± 0.3 **
16:4 ω-1	0.3 ± 0.01	0 *	1.1 ± 0.2	0 **
16:3 ω-4	22.5 ± 1.9	1.0 ± 0.01 **	21.0 ± 2.3	1.1 ± 0.2 **
16:2 ω-4	7.9 ± 0.1	0 **	6.4 ± 0.9	0 **
16:1 ω-7	23.5 ± 2.7	52.0 ± 0.1 **	28.6 ± 2.6	60.7 ± 2.3 **
16:0	12.2 ± 0.7	17.2 ± 0.3 **	11.4 ± 1.2	16.6 ± 0.3 *
18:4 ω-3	1.4 ± 0.1	1.2 ± 0.2	0.8 ± 0.1	0.9 ± 0.1
18:3 ω-3	0.1 ± 0.01	0.3 ± 0.01 **	0.1 ± 0.01	0.1 ± 0.001 *
18:2 ω-6	0.2 ± 0.03	1.0 ± 0.1 **	0.2 ± 0.003	0.4 ± 0.1
18:1 ω-9	0.4 ± 0.03	1.0 ± 0.05 **	0.5 ± 0.05	1.0 ± 0.1 *
18:0	0.8 ± 0.6	0.3 ± 0.03	0.19 ± 0.03	0 *
20:5 ω-3	23.3 ± 0.6	21.2 ± 0.2 *	19.4 ± 1.0	15.1 ± 1.7 *
22:6 ω-3	2.5 ± 0.3	1.9 ± 0.03	4.8 ± 0.3	1.6 ± 0.2 **
SFA (%TFA)	18.3 ± 0.03	20.4 ± 0.2 **	17.1 ± 1.3	19.2 ± 0.1
MUFA (%TFA)	23.9 ± 2.6	53.0 ± 0.2 **	29.1 ± 2.7	61.7 ± 0.2 **
PUFA (%TFA)	58.4 ± 3.2	26.6 ± 0.03 **	53.8 ± 3.9	19.2 ± 2.3 **

**Table 2:** EPA content, expressed as % DCW, production (mg L*^−^*^1^) and productivity (mg L*^−^*^1^ day*^−^*^1^), in a comparison between autotrophic and heterotrophic growth of *C. cryptica* at day 7 and day 14. Data are expressed as means ± SD, n = 3. Asterisks indicate significantly different values in autotrophic and heterotrophic conditions at *p* ≤ 0.05 (*) and *p*≤ 0.001 (**).

	EPA			
	Day 7		Day 14	
	Autotrophy	Heterotrophy	Autotrophy	Heterotrophy
Content (% DCW)	2.2 ± 2.5	2.2 ± 1.2	1.6 ± 1.5	2.7 ± 1.9 **
Yield (mg L^−1^)	7.3 ± 1.4	7.1 ± 1.0	12.2 ± 1.3	18.0 ± 0.7 **
Productivity (mg L^−1^ day^−1^)	1.0 ± 0.2	1.0 ± 0.1	0.9 ± 0.1	1.3 ± 0.1 *

**Table 3:** Comparison of biomass and EPA production of marine diatoms under heterotrophic conditions.

Algal Species	Volume (L)	Cultivation Period (days)	Glucose (g/L)	Biomass (g/L)	EPA (% DCW)	EPA (% in TFA)	Reference
*Cyclotella cryptica*	0.1	3	10	1	-	18–22	[30]
*Nitzschia laevis*	0.2	12	20	5.5	1.9	10.7	[41]
*Nitzschia laevis*	0.2	8	5	2.2	1.7	14.9	[24]
*Nitzschia laevis*	0.1	-	10	-	1.7	23.2	[23]
*Navicula incerta*	0.1	-	10	-	0.8	7.2	[23]
*Navicula pelliculosa*	0.1	-	10	-	0.5	4.6	[23]
*Cyclotella cryptica*	2	14	2	0.58	2.7	15.1	This study
